# Low Adherence to Mediterranean Diet Characterizes Metabolic Patients with Gastrointestinal Cancer

**DOI:** 10.3390/nu16050630

**Published:** 2024-02-24

**Authors:** Carlo De Matteis, Lucilla Crudele, Raffaella Maria Gadaleta, Ersilia Di Buduo, Fabio Novielli, Stefano Petruzzelli, Marica Cariello, Antonio Moschetta

**Affiliations:** 1 Department of Interdisciplinary Medicine, University of Bari “Aldo Moro”, 70124 Bari, Italy; carlo.dematteis@uniba.it (C.D.M.); lucilla.crudele@uniba.it (L.C.); raffaella.gadaleta@uniba.it (R.M.G.); ersiliadibuduo9704@gmail.com (E.D.B.); fabio.novielli@uniba.it (F.N.); stefano.petruzzelli@uniba.it (S.P.); 2 INBB National Institute for Biostructure and Biosystems, Viale delle Medaglie d’Oro 305, 00136 Rome, Italy

**Keywords:** gastrointestinal cancer, Mediterranean diet, Chrono Med Diet Score, nutrition

## Abstract

**Background.** Gastrointestinal (GI) cancers are one of the most relevant causes of death globally, frequently associated with poor dietary patterns. The Mediterranean Diet (MedDiet) contributes to cancer prevention. To assess adherence to MedDiet, our research group validated a new score, the Chrono Med Diet Score (CMDS), that captures increased visceral adiposity. **Methods.** We enrolled 401 subjects who underwent an evaluation for metabolic diseases and specific screening procedures according to current guidelines and were asked to answer CMDS. A total of 71 new cancer cases were recorded, including 40 GI and 31 non-gastrointestinal (NON-GI) cancers. **Results.** We found that CMDS was reduced in subjects who were diagnosed with cancers. Patients who reported a CMDS score of 12 or less had an over three times increased risk of being diagnosed with GI cancers and presented increased waist circumference and triglycerides and reduced HDL cholesterol compared to adherent subjects. **Conclusions.** Low CMDS values capture the risk for cancer diagnosis, especially for GI cancers. Thus, CMDS, along with waist circumference, can be considered as a bona fide marker for increased risk of cancer, requiring anticipated screening procedures for the detection of premalignant and early stage GI cancers in patients with low adherence to MedDiet.

## 1. Introduction

Gastrointestinal (GI) cancers include tumors of the colon, rectum, stomach, pancreas, esophagus, anus, gallbladder, liver, and bile duct and are among the top 10 causes of cancer morbidity and mortality worldwide [1].

Despite considerable advances in the understanding of the molecular pathogenesis of GI cancers, these malignancies still account for 6% of the global cancer incidence and 35% of all cancer-related deaths, especially in Western countries [2]. Most GI cancers develop over an extended period, representing an attractive opportunity for intervention and prevention strategies. Furthermore, a steady decline in the incidence of colorectal and gastric cancers was observed due to screening and diagnostic tools focused on the early detection of premalignant and early stage cancers [3]. However, these screening procedures do not yet target all subjects at risk and necessitate invasive and expensive procedures. Therefore, it is imperative to develop novel, non-invasive, and robust approaches that can facilitate the early detection and prevention of GI cancer.

Epidemiological studies consider individual and environmental characteristics to be the main modifiable risk factors for cancer. A high Body Mass Index (BMI) and visceral adiposity, alcohol consumption and smoking, a low intake of fruit and vegetables, and physical inactivity, together with chronic infections (i.e., Helicobacter pylori, hepatitis B, C, and Epstein–Barr virus) [4,5,6], represent the most important risk factors for promoting cancer development. Being overweight or obese increases the risk of at least 13 types of cancer, including GI cancers [7]. Alterations in insulin signaling, the role of oleic acid, dysregulation of adipose tissue-derived inflammation and hormonal pathways, and sex hormone metabolism are emerging as crucial links between obesity and cancer [8,9]. Responsibly managing nutrition and physical activity could prevent more than half of cancers occurring today [7], and the dynamic and complex cause–effect relationships between lifestyle and cancer should be evaluated in the framework of the individual’s exposome [10].

In this scenario, the Mediterranean Diet (MedDiet), which is based on a high intake of vegetables, fruit, legumes, non-refined cereals, and extra-virgin olive oil (EVOO); moderate consumption of fish; and a low intake of red meats can be considered an ideal healthy nutritional pattern for promoting longevity [11,12]. Many clinical and epidemiological studies have shown that high adherence to a MedDiet is correlated with a reduced risk of tumors [13,14,15,16] because of the antioxidant and anti-inflammatory nutrients contained in the MedDiet, which reduce cell degeneration and proliferation of cancer cells and protect the cell membrane from metastasis. EVOO, which is known for its high polyphenol content, can modulate the expression of several transcripts and miRNAs involved in numerous pathways, such as cell proliferation, inflammation, lipid metabolism, and cancer [17,18]. Moreover, the MedDiet has beneficial effects on the gut microbiota by promoting microbial diversity and preserving gut homeostasis and eubiosis [19]. Cancer development depends on several factors. Focusing on nutritional aspects, it is not possible to define which food participates in the prevention of cancer development. Since the MedDiet is more than a mere nutritional prescription, rather than a lifestyle embracing different aspects of life, culture, and traditions, [20] in recent years, different tools like a priori or a posteriori scoring of adherence systems [21] have been identified with the aim of assessing the whole diet and lifestyle pattern as levels of adherence to the MedDiet. This approach has consistently shown an inverse association between such dietary patterns and the overall mortality rate [22,23,24] in different pathological settings.

Our research group has recently developed a new, easy-to-use score, the Chrono Med Diet Score (CMDS), which considers lifestyle habits, in addition to information on food consumption already considered in previous scores such as the Med Diet Score (MDS), developed by Panagiotakos et al. to characterize adherence to the MedDiet [25]. Indeed, the CMDS includes eleven food categories, also considering the time of farinaceous product intake and physical activity. In the original study, we highlighted that CMDS is associated not only with MedDiet adherence but also with several conditions, such as visceral obesity, as evaluated by waist circumference (WC), dyslipidemia, glucose intolerance, increased cardiovascular risk, and liver steatosis [26].

In the present work, we show that in our study population, the CMDS, more than the MDS, is altered in patients who were diagnosed with GI cancers. Furthermore, we identify a CMDS cut-off that significantly distinguished patients with GI cancer, demonstrating that this score could be useful for identifying patients who should undergo prioritized screening procedures in consideration of their poor nutrition-related specific risk factors, thus representing an easy-to-use tool for personalized medicine.

## 2. Materials and Methods

### 2.1. Study Participants

Patients’ recruitment and clinical, anthropometric, and biochemical data were consecutively recorded in the electronic health registry for Metabolic Diseases of Department of Interdisciplinary Medicine—Internal Medicine Division of University of Bari “Aldo Moro” (Bari, Italy) from January 2020 to December 2023. A total of 908 outpatients (456 females, 452 males) were evaluated at our outpatient clinic to assess metabolic conditions. Among these, 401 subjects agreed to participate in this study (204 females, 197 males). All study participants underwent a first evaluation consisting of physical exam, biochemical evaluation, and abdominal ultrasound and answered specific questions about adherence to MedDiet and lifestyle through two different questionnaires, namely, CMDS and MDS, administered via standard operating procedures by trained personnel. Afterward, patients underwent screening procedures (i.e., colonoscopy, esophagogastroduodenoscopy, etc.) according to specific clinical requirements, current guidelines [27,28,29,30], and institutional procedures. A total of 71 cancer diagnoses were recorded, including a total of 40 GI cancers (28 colorectal, 7 hepatocellular carcinomas, 3 stomach, and 2 pancreas), with 31 cancers that were related to non-gastrointestinal (NON-GI) systems (11 thyroid, 10 prostate, 6 ovarian, and 4 endometrial). The study was approved by the Ethics Committee (n.311, MSC/PBMC/2015) of the Azienda-Ospedaliero-Universitaria Policlinico di Bari (Bari, Italy) in accordance with the principles of the Declaration of Helsinki [31]. Written informed consent for clinical data use was obtained from all participants. According to the Ethics Committee guidelines, only subjects who were already 18 years old or older at their first evaluation were included in the present study.

### 2.2. Clinical Assessment and Biochemical Measurements

Anthropometric assessment was performed using standardized procedures. Waist circumference (WC) was measured at the midpoint between the inferior part of the 12th costa and the anterior–superior iliac crest and was considered pathological according to the Metabolic Syndrome (MetS) 2006 International Diabetes Federation (IDF) definition of >94 cm in males and >80 cm in females [32]. Body Mass Index (BMI) was computed as weight (kg) divided by the square of height (m), and BMI values (kg/m^2^) of 25.0–29.9 and 30.0 were considered as overweight and obese, respectively [33]. The serum was processed after overnight fasting to assess biochemical markers for glucose and lipid metabolism.

### 2.3. Chrono MedDiet Score

The CMDS has been previously validated by our research group as a reliable score for evaluating adherence to the MedDiet, as well as its association with visceral adiposity [26]. Eleven food categories are considered to determine the overall score based on daily to weekly intake: (1) fruit, (2) vegetables, (3) legumes, (4) farinaceous products (i.e., bread, pasta, cookies), (5) grain cereals, (6) fish, (7) meat and meat products, (8) milk and dairy products, (9) olive oil, (10) butter, margarine and lard, and (11) alcohol intake. Two more categories were added to further characterize the eating habits and overall health status of the study participants: time of farinaceous product intake and physical activity. The overall score ranged from −13 to 25 points, which indicates the best adherence to this mixed rationale. We considered the validated cut-off of 13 points to indicate adherence to the MedDiet [26].

### 2.4. Med Diet Score

The MDS was developed by Panagiotakos et al. to characterize adherence to the MedDiet [25]. The overall score ranged from 0 to 55 points, with the latter indicating the highest adherence by assigning a score ranging from 0 to 5 for consumption of the same products included in the rationale of MedDiet. A specific category was dedicated to potato consumption, with a score of 5 points given for the recommended consumption of 3–4 portions per week, a score of 4 points given for 1–2 consumption per week, and lower scores for other frequency of consumption, weekly or daily. Alcohol intake was also considered, using wine glasses as a reference. A maximum score (5 points) was given for <3 wine glasses drank daily, no points were assigned for drinking >7 wine glasses, and scores ranging from 1 to 4 were assigned for the consumption of 3 to 7 wine glasses per day [25].

### 2.5. Statistical Analysis

Descriptive statistical analyses of the study sample were performed, and the results are expressed as mean ± standard deviation (SD) and frequencies (%) based on the considered variable. Comparisons of sociodemographic and clinical variables between two groups were conducted with the Student *t*-test for continuous variables. Receiver operating characteristic (ROC) curve was performed to identify the association between CMDS or MDS and GI cancer development. The area under the curve (AUC) was plotted to distinguish between clinical groups, and Youden’s Index, or equivalently, the highest Sensitivity + Specificity, was used to determine the optimal cut-off of each variable for the prediction of each condition. Pearson χ^2^ and Fisher’s exact tests were used for comparison of categorical variables. Results were expressed as Odds Ratios with their relative 95% confidence interval (95% CI) and graphically plotted in a forest plot. Correlation between continuous variables was analyzed using Spearman’s Correlation Coefficient (r). *p*-values lower than 0.05 were considered significant.

All statistical analyses were performed using NCSS 2023 Statistical Software, version 23.0.2 (NCSS, LLC, Kaysville, UT, USA) and GraphPad Prism, version 10.0.2 (GraphPad Software; San Diego, CA, USA).

## 3. Results

### 3.1. Baseline Characteristics of the Study Population

Mean baseline BMI (27.3 ± 4.3) and WC (96.0 ± 7.4) indicated mostly overweight patients, especially considering that mean WC was positive for MetS diagnosis according to IDF criteria. In Table 1, metabolic biomarkers like fasting plasma glucose (FPG), total cholesterol (TC), high-density lipoprotein cholesterol (HDL-c), low-density lipoprotein cholesterol (LDL-c), and triglycerides (TG) were not significantly altered. MedDiet adherence scores MDS and CMDS indicated an overall low adherence to the MedDiet.

### 3.2. Increased WC and Low HDL Cholesterol, Not BMI and LDL Cholesterol, Identify Cancer Patients

Cancer was identified during the clinical evaluation in a total population of 71 subjects (37 males, 34 females). We then divided our population according to cancer diagnosis, into two groups, to better understand the characteristics of both cancer patients and control subjects. As shown in Table 2, LDL-c did not differ between the two groups. On the other hand, WC (*p* = 0.0377), HDL-c (*p* = 0.0079), and FPG (*p* = 0.0175) were significantly altered in subjects who developed cancer, indicating that increased WC and low HDL cholesterol but not BMI or LDL cholesterol indicate an increased risk of cancer.

### 3.3. CMDS Is Associated with GI Cancer

To further analyze the potential association between cancer diagnosis and nutritional habits, we compared the values of CMDS and MDS in control and cancer subjects, as well as in GI cancers as opposed to NON-GI cancers. As shown in Table 3, CMDS was significantly altered in each subgroup that received a cancer diagnosis, whereas MDS was not. Indeed, this change in CMDS is striking (*p* = 0.0002), especially in patients who were diagnosed with GI cancers (6.3 ± 1.9), even when compared to subjects affected by other types of cancer (10.5 ± 3.1). MDS was not significant in any of the performed analyses, indicating a poor association with cancer diagnosis.

**Figure 1:**
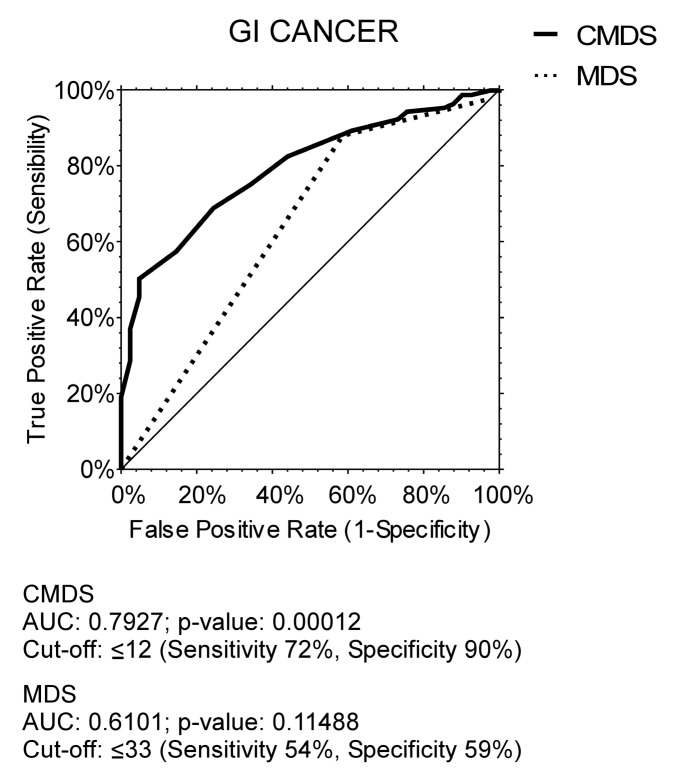
ROC curve of Chrono Med Diet Score and Med Diet Score for GI cancer diagnosis. Abbreviations: gastrointestinal cancer, GI cancer; Chrono Med Diet Score, CMDS; Mediterranean Diet Score, MDS; area under the curve, AUC.

Based on these results, to determine whether adherence scores could be accurately associated with GI cancer development, we performed ROC curves of CMDS and MDS (Figure 1) for GI cancer diagnosis. Results were significant regarding CMDS, with an AUC = 0.7927 (*p* = 0.0001) for GI cancer development, with a cut-off value of 12 (sensitivity = 0.72 and specificity = 0.9), whereas ROC for MDS was not significant with an AUC = 0.6101 (*p*= 0.11488).

**Figure 2:**
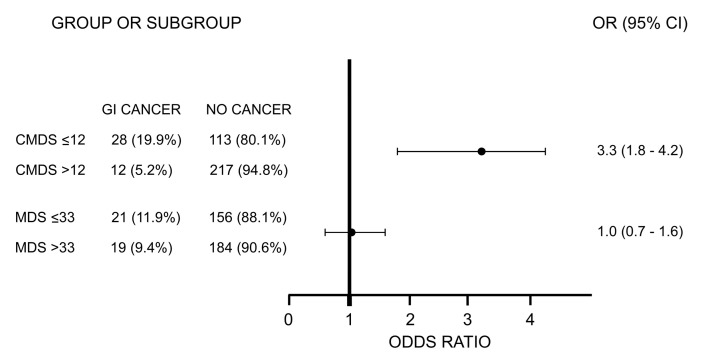
Odds Ratio of GI cancer association. Representation of Odds Ratio, showing the upper and lower limit of the confidence interval. Abbreviations: gastrointestinal cancer, GI cancer; Chrono Med Diet Score, CMDS; Med Diet Score, MDS.

To assess the association between GI cancer diagnosis and CMDS and MDS, we then calculated chi-square and OR (Figure 2). Intriguingly, subjects displaying a CMDS lower than the cut-off of 12 had a significant association with GI development (chi-square *p* = 0.0038). Thus, we calculated the OR finding that these subjects had an increased risk of developing GI cancer, with an OR of 3.3 (95% CI 1.8–4.2, *p* = 0.00033). In regard to MDS, the analysis still showed a significant OR, although of 1.0 (95% CI 0.7–1.6, *p* = 0.04886).

### 3.4. Characterization of GI Cancer Subjects According to CMDS Values

**Figure 3:**
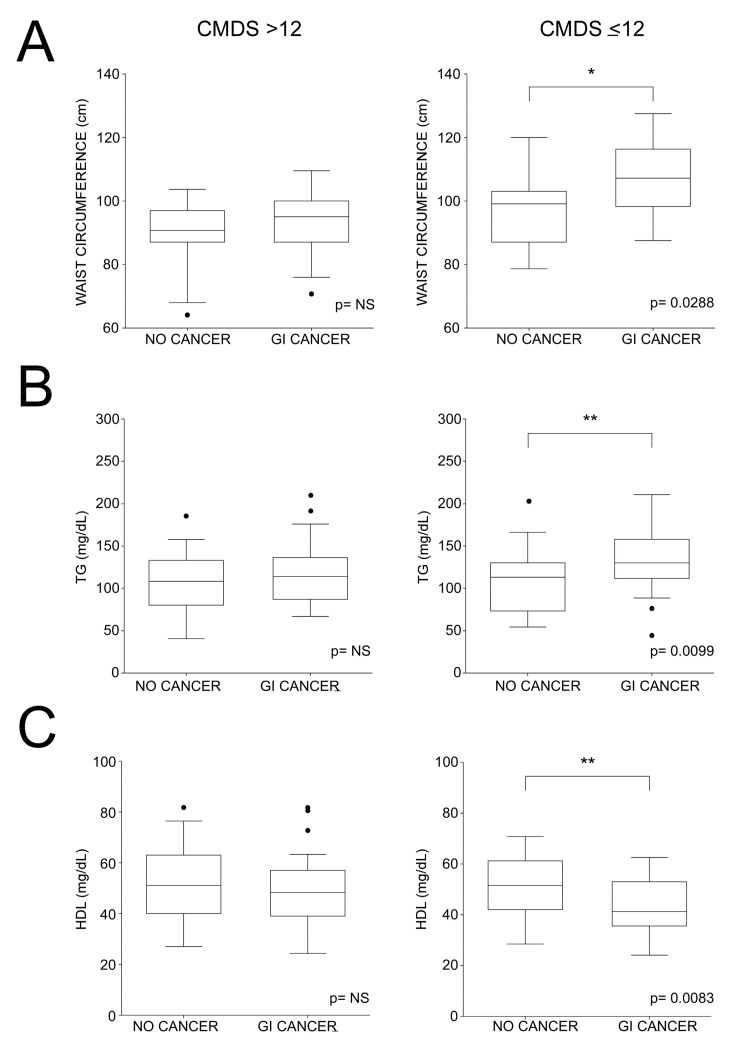
Comparisons of WC, triglycerides, and high-density lipoprotein cholesterol according to GI cancer diagnosis and Chrono Med Diet Score ROC identified cut-off for cancer association. Box plots show median (second quartile) and first and third quartiles. Tukey whiskers reach 1.5 times the interquartile distance or the highest or lowest point, whichever is shorter. Any data beyond whiskers are shown as black dots. Comparisons were performed using Students’ *t* test. (* *p* < 0.05; ** *p* < 0.01). (**A**) Box plots of waist circumference according to CMDS cut-off and GI cancer diagnosis; (**B**) Box plots of triglycerides according to CMDS cut-off and GI cancer diagnosis; (**C**) Box plots of glucose according to CMDS cut-off and GI cancer diagnosis; Abbreviations: gastrointestinal cancer, GI cancer; triglycerides, TG; high-density lipoprotein cholesterol, HDL-c.

Considering the significant association found between CMDS and GI cancer, we further analyzed the metabolic difference between subjects with GI cancer and subjects not affected by the disease. To better characterize the link between cancer and lifestyle, we performed a comparison in subjects with CMDS > 12 (Supplementary Table S1) and in subjects with CMDS ≤ 12 (Supplementary Table S2). In the first analysis, statistical significance was not achieved, as values of anthropometric indices such as WC and BMI did not differ between the two groups, highlighting the lack of differences in GI cancer diagnosis, although these patients were found to adhere to the MedDiet (n = 9), probably due to the incidence of cancer on a more genetically susceptible background. On the other hand, in patients with a CMDS ≤ 12, those who were diagnosed with GI cancer (n = 31) had significantly greater WC (106.2 ± 6.6, *p* = 0.0288) and TG levels (131.5 ± 17.1, *p* = 0.0099) compared to the other group (n = 129), while HDL-c levels were significantly lower (41.3 ± 7.4, *p* = 0.0083) (Figure 3A–C).

### 3.5. Correlations between GI Cancer Diagnosis and Specific CMDS Items

**Figure 4:**
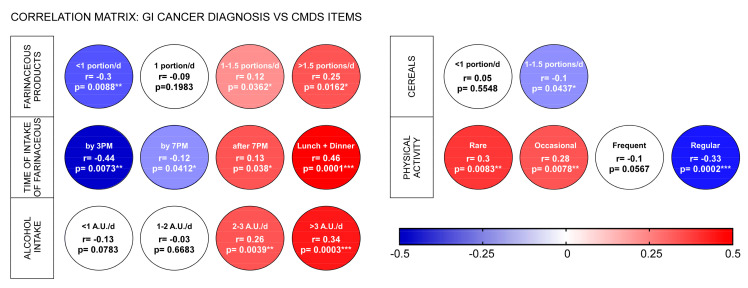
Correlation matrix of CMDS categories and GI cancer diagnosis in the overall population. Spearman correlations (r) between each CMDS category values and cancer diagnosis were performed. Non- significant correlations are shown as white circles. When correlation is significant (* *p* < 0.05; ** *p* < 0.01; *** *p* < 0.001), the circle is colored according to their value (see legend bar). Abbreviations: Alcohol unit, A.U.; day, d.

To assess the potential correlations between components of CMDS, like physical activity, carbohydrates (CHO) and their time of intake, cereals, alcohol intake, and the GI cancer diagnosis, we performed a correlation matrix, which showed that farinaceous products displayed a significant protective correlation (r = −0.33, *p* < 0.01) when intake was lower than 1 portion/day, while a more frequent consumption positively correlated with GI cancer diagnosis (r = 0.12, *p* < 0.05 for 1–1.5 portions/day and r = 0.25, *p* < 0.05 for more than 1.5 portions per day). The time of intake also strongly correlates with such a diagnosis. The consumption of CHO by 3PM was negatively correlated with GI cancer diagnosis (r = −0.44, *p* < 0.01), while, on the other hand, consumption at lunch and dinner was positively correlated with GI cancers (r = 0.46, *p* < 0.0001). Conversely, the correlations for cereals were not as strong as those for CHO, as only an intake of 1–1.5 portions/day was found to be significant (r = −0.1, *p* < 0.05). The physical activity frequency confirmed its protective role against cancer diagnosis (r = −0.33; *p* < 0.001 for more than 4 times per week), whilst rare (r = 0.3, *p* < 0.001) and occasional (r = 0.28, *p* < 0.01) physical activity were significantly correlated with GI cancer. Farinaceous products displayed a direct significant correlation with WC when the intake was higher than recommended (r = 0.311, *p* < 0.005 for >1.5 portions/die; r = 0.124, *p* < 0.05 for 1–1.5 portions/die) and a negative correlation when daily intake was lower (r = −0.292, *p* < 0.05 portion/die; r = −0.292, *p* < 0.05 for <1 portion/die). Additionally, the time of farinaceous products intake seems to play a role in this interplay since a direct significant association of this item with WC was found for lunch and dinner intake (r = 0.31, *p* < 0.0001) and after 7 pm (r = 0.22; *p* < 0.01), whilst a significant decrease in WC was detected when farinaceous were consumed by 3 pm (r = −0.41, *p* < 0.05). Regarding alcohol intake, results highlighted that alcohol consumption was positively correlated with GI cancer diagnosis for both moderate (r = 0.26, *p* < 0.005) and high (r = 0.34; *p* < 0.0005) consumption (Figure 4).

### 3.6. Correlations between CMDS and Serum Biomarkers

**Figure 5:**
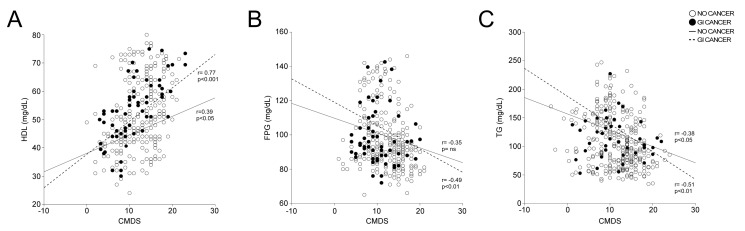
Correlation between Chrono Med Diet Score, HDL-c, FPG, and TG in GI cancer and no-cancer subjects. The correlation analysis was estimated using Spearman’s Correlation Coefficient (r); (p) indicates statistical significance. Each dot represents a single subject. (**A**) Correlation analysis of CMDS and HDL-c. (**B**) Correlation analysis of CMDS and FPG. (**C**) Correlation analysis of CMDS and TG. Abbreviations: Chrono Med Diet Score, CMDS; HDL cholesterol, HDL-c; Fasting Plasma Glucose, FPG; triglycerides, TG; gastrointestinal cancer, GI cancer.

Given the strong relationship between CMDS and GI cancer, especially regarding lipid biomarkers, we performed a correlation analysis between CMDS and levels of HDL-c, TG, and FPG. Intriguingly, we show that CMDS correlated with HDL-c levels in controls (r = 0.39, *p* < 0.05), but this correlation was even stronger (Figure 5A) in GI cancer subjects (r = 0.88, *p* < 0.001). CMDS was also negatively correlated with FPG (Figure 5B) in GI cancer subjects (r = −0.49, *p* < 0.01) but not in non-cancer subjects (r = −0.35, *p* = ns). Moreover, CMDS correlated with TG levels (Figure 5C), especially in GI cancer patients (r = −0.51, *p* < 0.01), but also in subjects not affected by cancer (r = −0.38, *p* < 0.05).

## 4. Discussion

In the present study, we found that patients reporting a CMDS score of 12 or less had an increased risk of developing GI cancers and presented increased WC and circulating TG and reduced HDL levels compared to control subjects. GI cancers, although significantly detected at earlier stages currently, still represent one of the most relevant causes of death globally, and colorectal cancer (CRC) alone is the third most frequently diagnosed cancer globally, with more than 3 million new cases and 1.5 million deaths expected to occur by 2040 [34,35]. We have previously demonstrated the role of low HDL-c levels as a bona fide novel marker to predict the development of hepatocellular carcinoma (HCC) in subjects with non-alcoholic fatty liver disease, as well as the importance of a high WC for such development [36]. Moreover, we identified specific features of metabolic dysfunction-associated liver disease (MASLD) as markers of thyroid and gynecological cancer risk, highlighting the importance of non-invasive, serum-based biomarkers for the early detection of such conditions and the role of healthy lifestyles in their prevention [37,38].

In our study, we showed that cancer patients had significantly greater WC and lower HDL-c than subjects in the control group. On the one hand, this confirms our hypothesis that visceral adiposity, through the production of hormones and cytokines, provides a fertile ground for cancer cells, proving the significant role of WC instead of BMI in metabolic-associated cancer development [6]. On the other hand, the role of HDL-c and the involvement of cholesterol pathways in cell proliferation have been confirmed, as cholesterol, via liver X receptor (LXR), a nuclear receptor and transcription factor involved in cholesterol homeostasis, is critical in inflammatory conditions, hence tumorigenesis, in a significant number of cancers [39,40]. The PREDIMED trial also provided evidence that a vegetable-based MedDiet, rich in unsaturated fat and polyphenols, can be considered an ideal model for cardiovascular disease (CVD) prevention, as significant positive changes in lipid profile, inflammation, blood pressure, and oxidative stress are all part of the MedDiet-associated anti-inflammatory effects and oxidative stress reductions [41].

In line with this, a robust association between adherence to the MedDiet and lower oxidative stress, as indicated by glutathione (GSH, reduced form) and glutathione disulfide (GSSG, oxidized form), was proven to further highlight the cardioprotective effects through the lowering of oxidative stress [42]. Nonetheless, as shown in the PREDIMED-Plus study, the role of MedDiet-induced modifications on the gut microbiota, as well as weight loss induced by an energy-restricted MedDiet and physical activity, may be explained by the increase in short-chain fatty acid-producing bacteria [43]. The consumption of specific nutrients also resulted in lower fecal bile acid concentrations and greater relative abundances of bacteria capable of fiber fermentation, providing evidence of diet-induced changes in the metabolic functions of the gut microbiota [44].

Recent extensive epidemiological data also support the positive correlation between CRC, a high-fat diet, and a sedentary lifestyle [45]. The MedDiet is universally considered to play a pivotal role in maintaining health status, reducing the risk of chronic disease development. Unfortunately, in many countries of the Mediterranean area, a progressive loss of adherence to MedDiet principles has been observed, endorsing the Western Diet model as an unhealthy diet with high consumption of processed fats and carbohydrates [46,47]. In this scenario, many MedDiet adherence scores have been generated and are often used for epidemiological studies or identification of subjects at risk of developing chronic degenerative diseases [48,49].

Very recently, aiming to identify increased risk for metabolic disease onset and development, we developed the CMDS, which significantly represents not only adherence to the MedDiet but also considers individuals’ lifestyles as a whole, capturing items such as the time of nutrient intake, sedentary lifestyle, and alcohol consumption that are crucial for the development of chronic conditions like cancer [26].

In this observational study, in 401 patients, we demonstrated that our CMDS was significantly lower at first evaluation in subjects who were diagnosed with cancer, especially GI cancers, whereas another significant a priori score for MedDiet adherence detection, MDS, did not identify this condition.

There is a large body of evidence to support the role of a MedDiet in preventing cancer at multiple organ sites [5,50], critically due to the intimate relationship between inflammation and carcinogenesis. Chronic inflammation causes DNA damage, mutations, and impaired DNA repair pathways, initiating and promoting cancer development [6,51,52]. All these issues have been addressed, among other strategies, by a balanced dietary lifestyle like the MedDiet. Nevertheless, the analysis of a single nutrient or group of nutrients represents a reductionist approach that cannot be considered sufficient for studies about the preventive effects of nutrition in chronic diseases such as cancer [53]. The European Prospective Investigation into Cancer and Nutrition (EPIC) study, which analyzed the relationship between MedDiet adherence and cancer in a population of 25,623 subjects with 7.9 years of follow-up, demonstrated that high MedDiet adherence was associated with a reduction in overall cancer incidence, particularly related to a 33% reduction in gastric cancer risk [13,54]. Morze et al. demonstrated an inverse correlation between gastric, bladder, and lung cancer incidence and adherence to MedDiet, as well as all-cause mortality among cancer survivors [55]. Furthermore, high adherence to a Mediterranean dietary pattern was inversely associated with the risk of colorectal, biliary tract, and pancreatic cancers [56,57,58]. The beneficial effects of a Mediterranean diet supplemented with extra-virgin olive oil were also shown in the primary prevention of breast cancer [59].

In line with these observations, our study demonstrated that a high MedDiet adherence is inversely correlated with cancer, especially GI cancers.

To characterize a Mediterranean eating pattern, Sofi et al. performed a meta-analysis that analyzed the effect of MedDiet interventions on multiple health outcomes (cardiovascular mortality, cancer incidence and mortality, and incidence of Parkinson’s and Alzheimer’s diseases), developing the MEDI-LITE score based on nine food categories [60,61]. Recently, Djuric et al. developed the Mediterranean Cancer Preventive Diet Score (MCAP) based on the traditional Mediterranean eating pattern and the American Institute for Cancer Research (AICR) and American Cancer Society (ACS) guidelines for cancer prevention [62]. This score confers positive points for seven preventive food categories, including Mediterranean fats, and negative points for four food categories associated with increased cancer risk, including ultra-processed foods [63]. The MCAP represents a useful score for epidemiological studies and for formulating dietary recommendations, but it has not yet been validated in a large cohort of patients. Here, we demonstrated that low adherence to the MedDiet, as evaluated via the CMDS, is associated with cancer, especially GI cancers, whereas MDS is not. Furthermore, we identified a cut-off value for discriminating patients with dysmetabolic conditions and an increased risk of developing GI cancers. The EPIC study revealed that increased WC was associated with an elevated risk of developing small intestine, gallbladder, stomach, esophageal, and colorectal cancer for both men and women, proving the close relationship between visceral obesity and GI cancers [8,64,65,66]. We have previously demonstrated that CMDS values equal to or less than 14 are strongly associated with increased visceral adiposity and liver steatosis [26]. In line with this, in this study, patients diagnosed with GI cancers who presented a CMDS ≤ 12 had increased WC and TG and reduced HDL, strongly indicating once again that this cut-off value characterizes dysmetabolic patients at greater risk of developing GI cancers. This study presents strengths and limitations that need to be addressed. First, screening procedures were conducted following the enrollment of each patient in the study. Therefore, other pre-existing conditions that contribute to cancer development could have played a significant role, along with an altered dietary pattern, in cancer development in each subject. Secondly, the lack of a gold standard and unified criteria to identify typical foods of the MedDiet implies that each score characterizes components based on regional criteria and available data. Regarding CMDS, we defined food groups according to previously validated, modern scores of adherence to this specific dietary pattern, and patients were asked to describe their eating habits with regard to such groups. Finally, the present study is an observational, association-based analysis of the potential role of the MedDiet in preventing cancer development, but further prospective longitudinal studies are required to determine the role of the CMDS in predicting and modulating the risk of cancer development, especially in young individuals.

A strength of our study, on the other hand, is that patients who were enrolled in the study were referred to our clinic for metabolic-related assessment and not for cancer diagnosis or specific nutritional diseases; therefore, these patients can be considered a good example of the general population, considering the obesity pandemic [66].

## 5. Conclusions

In conclusion, the results of the present study showed that a modern assessment of adherence to the MedDiet through the CMDS, also considering the time of specific food groups intake and physical activity, captures the increased risk of cancer diagnosis, especially GI cancers, differently from other scoring systems.

Considering our study population, MedDiet adherence, assessed through the CMDS, was significantly reduced in subjects who were diagnosed with cancer, being associated as well with a variety of dysmetabolic conditions that, taken all together, identified a more than three-fold increased risk of developing such conditions compared to that of adherent subjects. These results emphasize the role of a healthy lifestyle and nutrition in preventing dysmetabolic conditions that lead to cancer and, at the same time, should encourage clinicians to perform personalized screening strategies for patients with poor dietary habits and increased visceral adiposity.

## Figures and Tables

**Table 1:** Clinical characterization of the study population. Data are presented as mean ± Standard Deviation (SD). Abbreviations: Body Mass Index, BMI; waist circumference, WC; high-density lipoprotein cholesterol, HDL-c; low-density lipoprotein cholesterol, LDL-c; triglycerides, TG; fasting plasma glucose, FPG; Chrono Med Diet Score, CMDS; Med Diet Score, MDS.

Clinical Variable	N = 401 (197M:204F) Mean ± SD
Age (Years)	59.5 ± 5.9
BMI (kg/m^2^)	27.3 ± 4.3
WC (cm)	96.0 ± 7.4
TC (mg/dL)	177.4 ± 14.2
HDL-c (mg/dL)	52.5 ± 5.7
LDL-c (mg/dL)	105.2 ± 9.5
TG (mg/dL)	120.5 ± 13.5
FPG (mg/dL)	98.4 ± 11.4
CMDS	13.7 ± 1.9
MDS	33.0 ± 5.8

**Table 2:** Comparison between patients according to cancer diagnosis. Comparisons were performed with Student *t*-Test analysis. Data are presented as mean ± Standard Deviation (SD). (* *p* < 0.05; ** *p* < 0.01). Abbreviations: Body Mass Index, BMI; waist circumference, WC; total cholesterol, TC; high-density lipoprotein cholesterol, HDL-c; low-density lipoprotein cholesterol, LDL-c; triglycerides, TG; Fasting Plasma Glucose, FPG.

Clinical Variable	No Cancer N = 330 (160M:170F) Mean ± SD	Cancer N = 71 (37M:34F) Mean ± SD	*p*-Value
Age (Years)	61.7 ± 4.2	58.9 ± 5.8	0.3823
BMI (kg/m^2^)	26.8 ± 3.8	28.7 ± 4.4	0.2419
WC (cm)	94.1 ± 4.5	101.2 ± 6.8	0.0377 *
TC (mg/dL)	168.4 ± 12.3	191.5 ± 13.2	0.1102
HDL-c (mg/dL)	52.8 ± 7.4	46.1 ± 6.3	0.0079 **
LDL-c (mg/dL)	106.1 ± 11.1	106.4 ± 15.4	0.8582
TG (mg/dL)	111.3 ± 9.3	143.7 ± 21.9	0.1155
FPG (mg/dL)	92.4 ± 8.4	116.8 ± 6.8	0.0175 *

**Table 3:** Comparison of Chrono Med Diet Score (CMDS) and Med Diet Score (MDS) according to cancer and GI cancer diagnosis. Comparison was performed with Student *t*-Test analysis. Data are presented as mean ± Standard Deviation (SD). (*** *p* < 0.001). Abbreviations: Gastrointestinal cancer, GI cancer; non-Gastrointestinal cancer, NON-GI cancer.

	**No Cancer** **N = 330 (160M:170F)** **Mean ± SD**	**Cancer** **N = 71 (37M:34F)** **Mean ± SD**	***p*-value**
CMDS	14.9 ± 2.3	8.0 ± 2.1	0.0017 ***
MDS	36.1 ± 2.8	33.9 ± 5.8	0.2768
	**No Cancer** **N = 330 (160M:170F)** **Mean ± SD**	**GI Cancer** **N = 40 (27M:13F)** **Mean ± SD**	***p*-value**
CMDS	14.9 ± 2.3	6.3 ± 1.9	0.0029 ***
MDS	36.1 ± 2.8	33.4 ± 7.5	0.1882
	**GI Cancer** **N = 40 (27M:13F)** **Mean ± SD**	**Non-GI Cancer** **N = 31 (10M:21F)** **Mean ± SD**	***p*-value**
CMDS	6.3 ± 1.9	10.5 ± 3.1	0.0002 ***
MDS	33.4 ± 7.5	34.1 ± 6.9	0.8993

## Data Availability

Data presented in this study are available upon request from the corresponding author. The data are not publicly available due to ethical issues.

## Supplementary Materials

The following supporting information can be downloaded at: https://www.mdpi.com/article/10.3390/nu16050630/s1, Table S1: Comparison between patients according to GI cancer diagnosis and Chrono Med Diet Score ROC identified cut-off. Table S2: Comparison between patients according to GI cancer diagnosis and Chrono Med Diet Score ROC identified cut-off.
