# Supplementary material for: Low Adherence to Mediterranean Diet Characterizes Metabolic Patients with Gastrointestinal Cancer
Source: Nutrients. 2024 Feb 24;16(5):630. doi: 10.3390/nu16050630 (PMC10933917; doi:10.3390/nu16050630)
Supplement: Supplementary file 1 [file nutrients-16-00630-s001.zip › nutrients-2874613-supplementary.pdf]

Supplementary Table S1. Comparison between patients according to GI cancer diagnosis and Chrono Med Diet Score' ROC identified cut-off. Comparison was performed with Student T-Test analysis. Data are presented as mean ± SD (Standard Deviation). Abbreviations: Body Mass Index, BMI; Waist Circumference, WC; Total Cholesterol, TC; High-Density Lipoprotein Cholesterol, HDL-c; Low-Density Lipoprotein Cholesterol, LDL-c; Triglycerides, TG; Fasting Plasma Glucose, FPG.

**Clinical Variable**	**NO Cancer CMDS >12 N= 201 (90M:111F)**	**GI Cancer CMDS >12 N= 9 (7M:2F)**	**p-value**
Age (Years)	60.1±5.3	59.8±6.0	0.5564
BMI (Kg/m^2)	29.9±3.9	29.3±4.4	0.7839
WC (cm)	93.9±5.6	97.0±9.3	0.3382
TC (mg/dl)	177.5±6.0	183.2±17.4	0.3891
HDL-c (mg/dl)	52.0±5.7	47.4±8.4	0.1091
LDL-c (mg/dl)	97.4±7.3	100.5±18.3	0.6621
TG (mg/dl)	110.3±6.4	118.4±21.4	0.1182
FPG (mg/dl)	92.5±7.8	100.3±9.9	0.0929

Supplementary Table S2. Comparison between patients according to GI cancer diagnosis and Chrono Med Diet Score' ROC identified cut-off. Comparison was performed with Student T-Test analysis. Data are presented as mean ± SD (Standard Deviation). (**p*<0.05; ** *p*<0.01). Abbreviations: Body Mass Index, BMI; Waist Circumference, WC; Total Cholesterol, TC; High-Density Lipoprotein Cholesterol, HDL-c; Low-Density Lipoprotein Cholesterol, LDL-c; Triglycerides, TG; Fasting Plasma Glucose, FPG.

**Clinical Variable**	**No Cancer CMDS ≤12 N= 129 (70M:59F)**	**GI Cancer CMDS ≤12 N= 31 (20M:11F)**	**p-value**
Age (Years)	60.7±5.7	60.5±5.9	0.4112
BMI (Kg/m^2)	30.1±3.7	30.8±4.0	0.7663
WC (cm)	99.5±5.3	106.2±6.6	0.0288*
TC (mg/dl)	179.3±8.6	185.2±13.5	0.2843
HDL-c (mg/dl)	49.1±5.1	41.3±7.4	0.0083**
LDL-c (mg/dl)	102.8±11.4	106.2±11.5	0.2771
TG (mg/dl)	111.3±9.9	131.5±17.1	0.0099**
FPG (mg/dl)	100.3±5.8	110.4±8.6	0.0812
